# Enhancing detection of SARS-CoV-2 re-infections using longitudinal sero-monitoring: demonstration of a methodology in a cohort of people experiencing homelessness in Toronto, Canada

**DOI:** 10.1186/s12879-024-09013-9

**Published:** 2024-02-02

**Authors:** Lucie Richard, Rosane Nisenbaum, Karen Colwill, Sharmistha Mishra, Roya M. Dayam, Michael Liu, Cheryl Pedersen, Anne-Claude Gingras, Stephen W. Hwang

**Affiliations:** 1 MAP Centre for Urban Health Solutions, Unity Health Toronto, 30 Bond St, M5B1W8 Toronto, ON Canada; 2 Dalla Lana School of Public Health, University of Toronto, 155 College St, Toronto, Canada; 3 Sinai Health, Lunenfeld-Tanenbaum Research Institute, 600 University Ave, Toronto, ON Canada; 4 Department of Medicine, University of Toronto, 1 King’s College Circle, Toronto, Canada; 5 Harvard Medical School, 25 Shattuck St, Boston, MA USA; 6 Department of Molecular Genetics, University of Toronto, 1 King’s College Circle, Toronto, Canada

**Keywords:** SARS-CoV-2, Reinfection, Serological testing, COVID-19 testing, COVID-19, Homeless persons

## Abstract

**Background:**

Accurate estimation of SARS-CoV-2 re-infection is crucial to understanding the connection between infection burden and adverse outcomes. However, relying solely on PCR testing results in underreporting. We present a novel approach that includes longitudinal serologic data, and compared it against testing alone among people experiencing homelessness.

**Methods:**

We recruited 736 individuals experiencing homelessness in Toronto, Canada, between June and September 2021. Participants completed surveys and provided saliva and blood serology samples every three months over 12 months of follow-up. Re-infections were defined as: positive PCR or rapid antigen test (RAT) results > 90 days after initial infection; new serologic evidence of infection among individuals with previous infection who sero-reverted; or increases in anti-nucleocapsid in seropositive individuals whose levels had begun to decrease.

**Results:**

Among 381 participants at risk, we detected 37 re-infections through PCR/RAT and 98 re-infections through longitudinal serology. The comprehensive method identified 37.4 re-infection events per 100 person-years, more than four-fold more than the rate detected through PCR/RAT alone (9.0 events/100 person-years). Almost all test-confirmed re-infections (85%) were also detectable by longitudinal serology.

**Conclusions:**

Longitudinal serology significantly enhances the detection of SARS-CoV-2 re-infections. Our findings underscore the importance and value of combining data sources for effective research and public health surveillance.

**Supplementary Information:**

The online version contains supplementary material available at 10.1186/s12879-024-09013-9.

## Background

Asymptomatic SARS-CoV-2 infection is common [1]. Thus, measurement techniques that minimize undercount and bias [2] are critical for fully understanding how COVID-19 infection is linked to downstream health outcomes, such as post-COVID condition, and to what degree public health policies and interventions have been effective. Now that variants capable of evading prior immunity have emerged and proliferated, measurement of COVID-19 burden must also consider the burden associated with SARS-CoV-2 re-infection.

Currently, most studies that measure SARS-CoV-2 re-infection define it using positive PCR tests more than 90 days apart [3]. Reports using this method suggest that re-infections are relatively rare, even after the emergence of the Omicron BA.1 variant [3–5]. However, this flies against the public perception of COVID-19 re-infection being common (for example, Pelley 2022 [6]). Some have speculated that the low re-infection rates reported in the literature to date may reflect re-infection definitions that require optimal testing conditions (such as regular PCR testing) not typically available in wider population-based studies [7, 8]. Indeed, relying solely on community-based PCR test surveillance is known to introduce undercount as well as potential measurement bias in settings where testing availability and accessibility vary [2, 9–11]. This is why COVID-19 studies have often leveraged serologic assay data to measure antibody levels against SARS-CoV-2 infection [9–11], which is accepted by the US Centers for Disease Control and Prevention (CDC) as “supportive evidence” of infection in its revised laboratory diagnostic criteria [12].

Given this, it is surprising that PCR testing alone remains widely accepted as a measure of SARS-CoV-2 re-infection, despite the undercount and bias issues that may result from this methodology. In this study, we use data from a prospective cohort study of people experiencing homelessness in Toronto, Canada, to introduce a novel approach that combines longitudinal serology and testing data to comprehensively identify SARS-CoV-2 re-infection. Using this method, we assess the existence and extent of SARS-CoV-2 re-infection undercount using PCR test data alone, assess the strengths and limitations of longitudinal serology for this outcome and calculate the rate of SARS-CoV-2 re-infection in a population of people experiencing homelessness.

## Methods

### Setting and design

This longitudinal analysis uses data collected between June 2021 and October 2022 from participants of the *Ku-gaa-gii pimitizi-win* study, a prospective cohort study of people experiencing homelessness in Toronto, a city on Treaty 13 territory in Canada. The *Ku-gaa-gii pimitizi-win* study protocol is available elsewhere [13].

At the time of recruitment (June to September 2021), the Delta variant (B.1.617.2) had largely replaced the Alpha variant (B.1.1.7) in COVID-19 infections in Toronto [14]. During this period, people experiencing homelessness were demonstrated to have a higher COVID-19 infection burden as compared to housed counterparts [15]. As a result, this population was prioritized for COVID-19 vaccination, and through substantial outreach efforts, uptake by people experiencing homelessness by the recruitment period was excellent (> 80%), approximating that of their housed counterparts in the region [16]. Throughout the observation period, non-pharmaceutical public health measures (such as masking and physical distancing) were also in widespread use.

In December 2021, the Omicron variant BA.1 replaced Delta, increasing from < 1% to > 95% of infections over the month [17]. Since then, variants increasingly capable of evading prior immunity have predominated in large waves of activity throughout 2022 [14], with people experiencing homelessness continuing to experience high rates of SARS-CoV-2 infection [18].

### Recruitment and follow-up

Participant recruitment and sample size calculation are described in detail in the study protocol [13]. Briefly, we recruited participants by random number schedule from beds, rooms or tents at 62 participating shelters, physical distancing hotels, and urban encampments between June 16 and September 9, 2021. Participants completed a baseline data collection interview that covered the period from March 1, 2020, to their interview date, and they were re-contacted for follow-up at three, six, nine, and twelve months (+/- 45 days). Data from Indigenous participants are owned by Anishnawbe Health Toronto, and were not included in this analysis.

At each interview, participants completed a survey detailing self-reported positive PCR or rapid antigen test (RAT) tests and COVID-19 vaccination events during the period between the last interview and the current interview. Participants also provided saliva (swish and gargle method) and blood samples (plasma tube [BD365985] and/or dried blood spots [Whatman 903]) to test for current SARS-CoV-2 infection and past SARS-CoV-2 infection/vaccination, respectively. The saliva sample was tested using standard quantitative reverse transcription polymerase chain reaction (RT-qPCR) [19], while the blood sample was analyzed using a plate-based enzyme-linked immunosorbent assay [20], measuring IgG antibodies to spike protein trimer (‘anti-S’), receptor-binding domain protein (‘anti-R’), and nucleocapsid protein (‘anti-N’). At 99% specificity, the sensitivity for plasma or serum samples is 94%, 89%, and 79% for anti-S, anti-R and anti-N, respectively; for dried blood spot, it is 98% for anti-S and anti-R and 92% for anti-N (more information about the assay performance characteristics are available elsewhere [20]). Relative ratios were calculated by dividing the raw value (luminescence counts per second) of each sample at a given dilution by a blank-subtracted mid-point value of a recombinant antibody standard (0.0156 µg/mL of VHH72hFc1 × 7 (National Research Council of Canada) for spike and receptor-binding domain protein (RBD), 0.0156 µg/mL and 0.03126 µg/mL of anti-N HC2003 (Genscript Cat #A02039) for N) that was included as a control in each plate [20].

To ensure participants were at risk of re-infection at some point during the observation window, this analysis included participants with incident SARS-CoV-2 infection by the 9-month interview. Incident SARS-CoV-2 infection was determined through any of the following: (a) self-reported positive PCR or RAT test; (b) positive RT-qPCR test administered during the interview; or (c) newly positive serology. Seropositivity was defined in non-vaccinated individuals as having antibodies to at least two of three anti-SARS-CoV-2 antigens which exceeded positivity thresholds at the primary dilution [1:160 (0.0625µL/well) for plasma samples; and 1:4 (2.5µL/well) for dried blood spot] (see Supplementary Materials). Among vaccinated participants, a positive anti-N protein signal was required to support a SARS-CoV-2 infection, as COVID-19 vaccines approved in Canada increase anti-R and anti-S levels and thus cannot be used as a surrogate infection measure.

### Outcomes

Our outcome of interest was SARS-CoV-2 re-infection, which we defined both through standard test-based methods as well as through our novel approach combining longitudinal serology and testing data (herein referred to as ‘comprehensive method’ for brevity). We defined re-infection for the test-based method as any positive PCR or RAT test result (self-reported or study administered) occurring more than 90 days after the incident SARS-CoV-2 infection. We selected a 90-day cut-off following CDC recommendations [12] and most existing literature [3].

By contrast, the comprehensive method includes re-infections identified using the aforementioned test-based definition but also adds re-infections identified through changes observed in serologic assay data from follow-up interviews. To summarize, we identified re-infections among participants with sero-reverted anti-N levels whose anti-N levels subsequently increased above the positivity threshold. We also identified re-infections among participants with seropositive anti-N levels that increased above the assay’s coefficient of variation (new anti-N value > old anti-N value*1.294) if a downward trend in anti-N levels had been previously established. Supplement 1 provides a step-by-step procedure and flowchart detailing how the longitudinal serology data was used to identify re-infections.

The method for identifying re-infections using longitudinal serology applies current evidence that anti-N levels steadily decrease after an initial peak following infection [21, 22]. Our method is based on an assumption that, when the anti-N signal is already seropositive and showing a pattern of decrease over time, any new increase beyond the assay’s coefficient of variation is sufficient to indicate a new SARS-CoV-2 infection. As individuals mount highly variable anti-N responses, particularly in highly vaccinated environments [23, 24], it is not possible to link a particular amplitude of change in anti-N levels with a probability of infection. Furthermore, a recent study assessing change in anti-N as a means of improving identification of SARS-CoV-2 infection [25] found that increases only slightly above the coefficient of variation were optimal to ensure comprehensive capture of infection without including false positives. Thus, an increase above the coefficient of variation representing evidence of possible re-infection was deemed a reasonable working assumption until further validation work can be undertaken.

Finally, we categorized participants with serology-identified re-infection according to the level of supporting anti-R or anti-S antigen evidence available. Re-infections were deemed to be *probable* where concurrent, elevated anti-R or anti-S were observed among participants without recent vaccination; *possible* where concurrent, stable or elevated anti-R or anti-S were observed among recently vaccinated participants; or *indeterminate* where concurrent, decreasing anti-R or anti-S levels were observed, irrespective of recent vaccination.

### Statistical analysis

We provide counts and rates per 100 person-years for re-infections identified through test-only, serology-only and the comprehensive method over the pandemic (from March 1, 2020, to the final interview), over the 12 months of follow-up, and at each interview. Person-time was calculated from the date of incident infection onwards. Where incident infection or re-infection was identified through serology only, we assigned to the infection a random onset date between the previous and current interview dates, or within one year of the baseline interview for cases occurring prior to baseline. In all instances where a full 12-month follow-up was not achieved, participants were censored at their final interview to calculate rates by person-time at risk (for example, we consider participants who only provided baseline data for March 1, 2020, to baseline interview period only). 95% confidence intervals (CI) for re-infection rates per 100 person-years were calculated using Poisson regression.

We also assessed concordance between the test-based method and the same or subsequent period’s serology results (depending on the date of infection onset, antibodies might only be mounted by the subsequent interview sample). This allows us to assess potential misclassification introduced by the longitudinal serology data and highlight situations in which serology-only methods may be unable to identify re-infection.

Finally, for descriptive purposes and to further assess concordance between testing and serology data, we provided summary measures comparing test-identified re-infections, serology-identified re-infections, and interviews subsequent to incident infection but without evidence of re-infection (randomly selecting an interview following the distribution of interviews with evidence of re-infection).

All analyses were conducted using SAS enterprise guide v7.1 (SAS Institute Inc., Cary, NC, USA).

### Ethical review

This study received ethics approval (REB# 20–272 and 20-0266-E) from the Research Ethics Board at Unity Health Toronto and Mount Sinai Hospital.

## Results

**Fig. 1:**
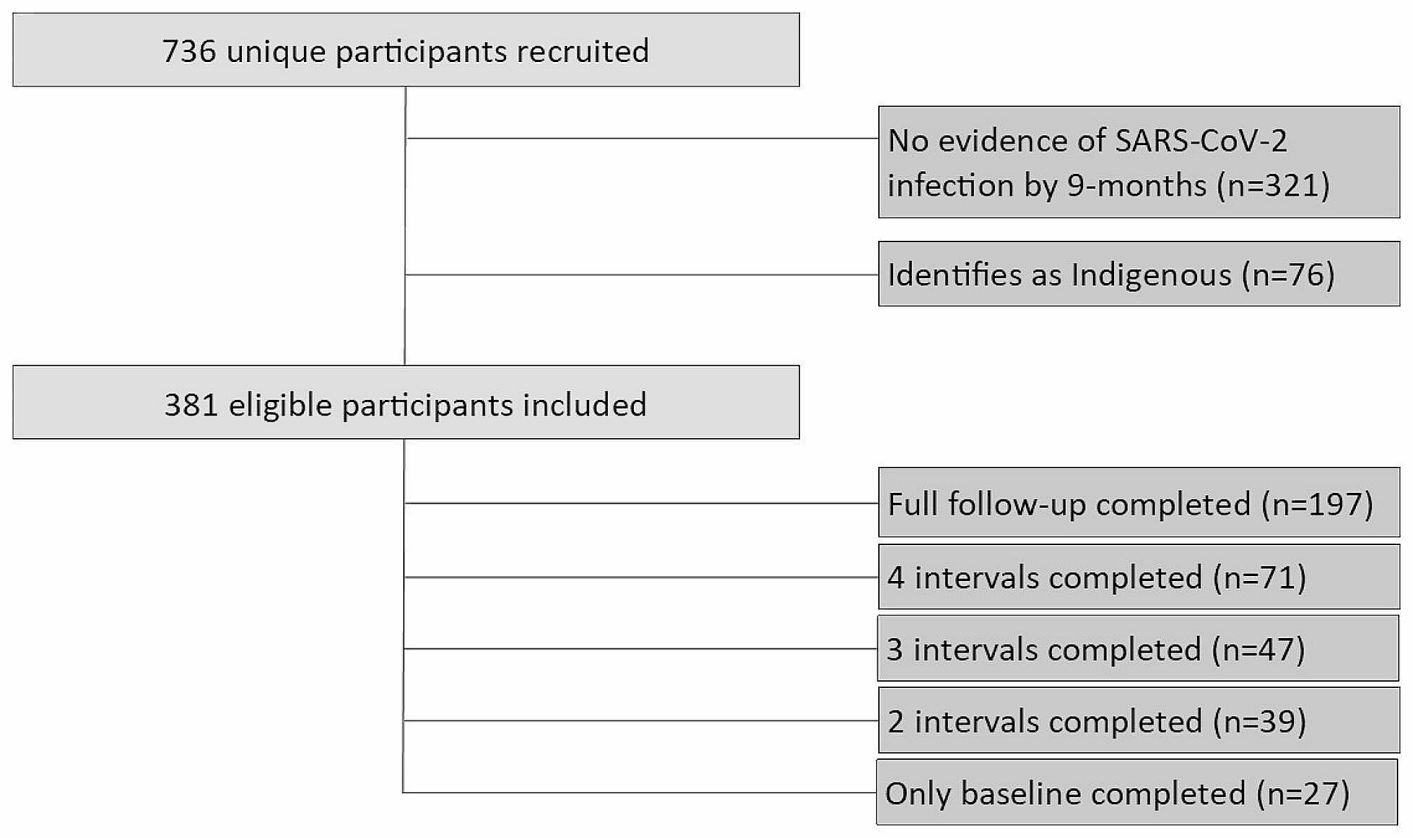
Ku-gaa-gii pimitizi-win recruitment, reasons for non-participation and degree of follow-up achieved

We included 381 eligible participants who experienced incident infection by their 9-month interview (Fig. 1). Of these, 197 participants had complete follow-up; 71 participants provided four intervals; 47 provided three intervals; 39 provided two intervals; and 27 participants had no follow-up information. For participants with only baseline interview information, re-infections could be identified using test-only methods.

Characteristics of participants at baseline are presented in Supplement 2. Briefly, participants had a mean age of 46.7 years (SD 15.4 years). Most participants were male (69.3%), Canadian citizens (71.4%), and received a full course of COVID-19 vaccination by the baseline interview (59.9%). The recruitment period preceded the widespread availability of booster doses.

Re-infections identified through test-only (PCR and/or RAT), serology-only and the comprehensive method combining both are summarized in Table 1 over all periods of interest. We identified 37 re-infections using the test-only method (33 from PCR; 4 from RATs) between March 1, 2020, and the final interview, representing 8.7 events per 100 person-years at risk. Of these, 31 re-infections occurred during the 12-month observation period. A further 98 re-infection events could only be identified using longitudinal serology, representing an additional 28.4 events per 100 person-years at risk. Together, our comprehensive method identified a total of 129 potential re-infection events over the 12-month observation period, averaging 37.4 events per 100 person-years at risk. The person-time rate varied widely by interview, with the highest rates occurring after Omicron became dominant in Toronto (80.5, 72.9, and 41.6 re-infections per 100 person-years at 6, 9, and 12 months, respectively).

**Table 1:** Number and rate per 100 person-years at risk of SARS-CoV-2 re-infections among Ku-gaa-gii pimitizi-win participants having evidence of incident infection (*n* = 381), by method of identification and observation period

	Test-only method		Longitudinal serology					Comprehensive method	
Period of Interest	*N*	Rate/100 YAR (95% CI*)	*N* Pro^a^	*N* Po^b^	*N* Ind^c^	*N* total	Rate/100 YAR (95% CI*)	*N*	Rate/100 YAR (95% CI*)
March 2020 to final interview (*n* = 381)	37	8.71 (6.2–11.9)	NA	NA	NA	NA	NA	135	31.78 (26.8–37.5)
Baseline interview to final interview (*n* = 381)	31	8.98 (6.2–12.6)	21	40	37	98	28.37 (23.2–34.4)	129	37.35 (31.3–44.2)
Specific periods:									
March 2020 to baseline Interview (*n* = 202)	6	5.74 (2.3–11.9)	NA	NA	NA	NA	NA	6	5.74 (2.3–11.9)
Baseline interview to 3-mth interview (*n* = 221)	0	0 (0–0)	0	3	1	4	9.33 (3.0-22.5)	4	9.33 (3.0-22.5)
Most recent interview (baseline or 3mth) to 6-month interview (*n* = 318)	12	21.47 (11.6–36.5)	8	19	6	33	59.05 (41.3–82.0)	45	80.52 (59.4-106.8)
Most recent interview (baseline, 3- or 6-mth) to 9-month interview (*n* = 381)	12	17.86 (9.7–30.4)	7	13	17	37	55.06 (39.3–75.1)	49	72.92 (54.5–95.6)
Most recent interview (baseline, 3-, 6-, or 9-mth) to 12-month interview (*n* = 381)	7	9.34 (4.1–18.5)	6	5	13	24	32.02 (21.0-46.9)	31	41.36 (28.6–58.0)

CI = Confidence Interval; YAR = Years at risk

*95% confidence interval calculated using Poisson regression

^a^Pro=Probable re-infection = Serology-identified re-infections with supporting anti-R or anti-S evidence unexplained by recent vaccination

^b^Po=Possible re-infection = Serology-identified re-infections with stable or supporting anti-R or anti-S evidence with recent vaccination

^c^Ind=Indeterminate re-infection = Serology-identified re-infections without supporting anti-R or anti-S evidence

Table 2 presents the relevant serology results for the 31 re-infections identified through testing after baseline. In 22 instances (or 85% of test-identified re-infections having at least one additional interview of follow-up), longitudinal serology also identified the re-infection during the same or subsequent interview, with most cases (*n* = 16) lacking concurrent anti-R or anti-S evidence (‘Possible’ or ‘Indeterminate’ re-infections). In five cases, we had insufficient information to deem the case concordant or discordant, mostly because the PCR test occurred on or within 14 days of the final interview, which may not have provided sufficient time to mount an antibody response. The four discordant cases can be broken down as follows: Three participants mounted no anti-N response (all three were fully vaccinated with boosters within the previous 6 months); and one participant’s anti-N RR values were above the linear range during both interviews and at both titers, making it impossible for our method to detect meaningful increases.

**Table 2:** – Comparison of serology and PCR/RAT evidence among participants with first or second re-infections identified through PCR or RAT testing (*N* = 31)

	Number (%)
**Agreement**	
Serology=’*Probable*’ same or next interview	6 (19.3%)
Serology=*’Possible*’ same or next interview	7 (22.6%)
Serology=’*Indeterminate’* same or next interview	9 (29.0%)
**Missing data, cannot determine agreement**	
+PCR test < 14 days before final interview^a^	4 (12.9%)
Self-reported + PCR test > 4 months before next interview^b^	1 (3.2%)
**Disagreement**	
Method unable to detect: both N values above linear range at both dilutions	1 (3.2%)
No evidence, no explanation	3 (9.7%)

^a^ These participants might have mounted antibodies by the subsequent interview but had no further interviews

^b^ This participant missed a few interviews and reported a positive PCR test early in this prolonged period. It is possible they mounted a response which cleared by the time serology was again available

Table 3 summarizes anti-N level characteristics in interviews during and immediately preceding evidence of a first re-infection (by evidence type), as compared to randomly selected interviews following incident infection for participants who never had evidence of re-infection. Overall, results were much more similar between re-infection types than between re-infections and interviews without evidence of re-infection. Interviews immediately preceding re-infection events had lower levels of anti-N than interviews for participants without evidence of re-infection (median 0.45 [testing] and 0.40 RR [serology only] vs. 0.64 RR) and had higher anti-N during the re-infection interview (median 1.23 and 2.31 RR vs. 0.56 RR). They also had much higher differences and ratios in anti-N between interviews (median difference 0.58 and 1.29 RR vs. -0.14 RR; median ratio 5.93 and 6.21 RR vs. 1.45 RR). The average anti-N level during re-infection interviews identified by serology was beyond the maximum of the linear range (2.0 RR), suggesting the true RR level is underestimated. Finally, on average, nearly a year (and most of the time more than one interview) passed between incident infection and re-infection, with re-infections identified through serology identified slightly sooner than test-identified re-infections (mean 322.82 days vs. 360.97 days).

**Table 3:** Summary characteristics of Antibodies to Nucleocapsid protein (anti-N) during first re-infection events occurring during the observation window (*n* = 118 of *n* = 129 re-infection events overall), by identification method, vs. a randomly selected interview following incident infection and without evidence of re-infection (*n* = 200)

		Random interview without evidence of re-infection (*N* = 200)	First re-infection event	
			**Identified through longitudinal serology only (** *N* ** = 90)**	**Identified through testing (** *N* ** = 28)**
*Anti-N RR* ^*a*^ *in previous interview*	Mean (SD) RR	1.06 (1.08)	0.70 (0.74)	0.64 (0.85)
	Median (IQR) RR	0.64 (0.39–1.57)	0.40 (0.24–0.84)	0.45 (0.13–0.69)
*Anti-N RR* ^*a*^ *during interview*	Mean (SD) RR	0.9 (0.92)	2.32* (1.39)	1.81 (1.61)
	Median (IQR) RR	0.56 (0.3–1.17)	2.31* (0.94–3.49*)	1.23 (0.40–3.31*)
*Difference* ^*b*^ *in anti-N RR* ^*a*^ *between interviews*	Mean (SD) RR	-0.19 (0.88)	1.62 (1.21)	1.16 (1.60)
	Median (IQR) RR	-0.14 (-0.44–0.03)	1.29 (0.48–2.79*)	0.58 (-0.08–2.67*)
*Ratio* ^*c*^ *in anti-N RR* ^*a*^ *between interviews*	Mean (SD) RR	1.45 (3.74)	6.21 (6.89)	5.93 (8.19)
	Median (IQR) RR	0.79 (0.57–1.12)	3.64 (1.99–7.18)	3.17 (0.86–6.62)
Days between infections	Mean (SD)	N/A	322.82 (147.50)	360.97 (142.10)
	Median (IQR)	N/A	315.94 (209.40–414.00)	338.57 (280.30–448.00)

Anti-N = Antibodies to Nucleocapsid protein; RR = Relative Ratio; IQR = Interquartile Range

^a^Anti-N relative ratios are derived from the blood sample (plasma or dried blood spot) ELISA, primary dilution (1:160 (0.0625µL/well) only

^b^Difference in anti-N RR represents [anti-N RR at selected interview– anti-N RR at immediately preceding interview]

^c^Ratio in anti-N RR represents [anti-N RR at selected interview / anti-N RR at immediately preceding interview]

*Value is outline of the linear range of the assay: results are thus likely an underestimate of true levels

## Discussion

We identified a substantial number of SARS-CoV-2 re-infections in our cohort of people experiencing homelessness. The rate of re-infections identified through testing alone (8.71 events per 100 person-years at risk) greatly exceeds pooled estimates (approximately 3.31 and 2.55 events per 100 person-years, respectively) from recent syntheses of SARS-CoV-2 re-infection studies, even those limited to post-Omicron periods [3, 26]. This corroborates a recent study which suggests that people experiencing homelessness may have a heightened risk for re-infection compared to housed counterparts [27].

In addition to test-identified re-infections, we detected 98 re-infections by analyzing trends in longitudinal serological data over the follow-up period. Including these additional cases, the overall rate of re-infections identified using our comprehensive method is 37.4 events per 100 person-years, more than four-fold higher than the rate from test-only methods.

Several auxiliary findings support the credibility of these serology-identified re-infections. First, the low rate of identification for testing-based re-infections is consistent with our previous findings on incident infections [18], where only 28% of incident infections were detectable using PCR or RAT. Second, the majority (85%) of test-identified re-infections with sufficient serology follow-up had supporting serology evidence, including instances where anti-N levels remained seropositive. Third, the average time between infections approached a year (similar to test-based methods), and most often occurred across a number of interviews, reducing the risk of misclassifying long-lasting infection episodes as re-infections. Fourth, changes in anti-N levels far exceeding the antigen’s coefficient of variation were observed in most re-infection events, greatly reducing the risk that random noise could have been misinterpreted as a re-infection event. Fifth and finally, a similar strategy of using repeated samples to assess change in anti-N data was used to improve serology identification of SARS-CoV-2 infections among vaccinated blood donors with PCR-confirmed infections [25]. Of note, the value this study deemed necessary to comprehensively identify infections without including false positives was only slightly above their assay’s coefficient of variation, suggesting that our approach of including seropositive increases above the coefficient of variation after a downward trend is reasonable and, indeed, necessary [25]. Collectively, these findings strongly support the validity of these serology-identified re-infections, despite the absence of conventional PCR or genomic evidence typically used for identification.

We did find a substantial number of re-infections unsupported by changes in anti-R or anti-S (‘indeterminate’). Although we cannot rule out false positives for anti-N, the number of indeterminate cases (including several among cases confirmed by testing) suggests it is more likely that the ELISA assay, which uses wildtype spike and RBD as antigens, may not have captured antibodies specific to certain variants of concern, particularly Omicron variants. Furthermore, because many ‘indeterminate’ cases had relatively high levels of anti-S and anti-R (above 1.0 RR), it is also possible that already circulating anti-S and anti-R levels (which are known to persist much longer than anti-N [28, 29]) were sufficient to clear the infection without new antibodies being generated. Because of the above, we consider ‘indeterminate’ re-infections to be equally credible to those labelled ‘probable’ and ‘possible’.

Undercounting re-infections when conducting research about COVID-19 is problematic. For example, in our recent study examining factors associated with incident SARS-CoV-2 infection among people experiencing homelessness [18], we found no significant association between housing-related factors and infection risk, challenging previous predictions [30, 31]. This unexpected result could be partly attributed to the definition of SARS-CoV-2 incidence used (incident infection), which may have been too insensitive in a cohort with a high prevalence of past infection. In future work, comprehensive capture of re-infection will also be essential to understanding whether and how infection burden (again, measured as the number of infections) relates to adverse outcomes. SARS-CoV-2 re-infection has been tied to increased risk of death, hospitalizations and other health sequelae [32], but it is unclear how the use of a comprehensive definition of re-infection might alter the magnitude of these risks.

Altogether, our findings suggest that SARS-CoV-2 re-infection rates reported in the literature to date may be significantly underestimating true COVID-19 infection burden, and longitudinal sero-monitoring offers a valuable opportunity to improve the detection of SARS-CoV-2 re-infection. This approach avoids excessive inconvenience to research participants compared to frequent PCR testing. It also reduces reliance on community surveillance, which is prone to bias [2] in places where PCR testing is not widely available and/or accessible, such as in Ontario, Canada where PCR testing was dropped as a public health surveillance strategy in late 2021.

### Limitations

Although our study benefits from data collected longitudinally among a randomly sampled group, participants could opt to cease participation or miss a follow-up period. Censoring and gaps between interviews may have reduced our ability to identify re-infections. Additionally, although participants received PCR testing at each interview, most of the test evidence was self-reported. While reports occurred every three months, helping to prevent issues of increasing unreliability over time, self-report data can suffer from social desirability bias, particularly among populations facing significant stigma. This could also have contributed to an undercount in both infection and re-infection rates. Finally, the serology method, which identified most of the re-infections in our comprehensive method, is not yet validated. Our preliminary evaluation indicates these re-infections seem credible. Nevertheless, future work should validate this method against a gold standard of known re-infection status, for example a cohort with highly frequent PCR test screening.

## Conclusions

A large number of SARS-CoV-2 re-infections can be identified using longitudinal sero-monitoring, indicating re-infections may be much more common than currently reported in the literature using current PCR test-based methods. The accurate and comprehensive measurement of SARS-CoV-2 re-infection is vital for future COVID-19 research. While a highly specific definition of re-infection is desirable to prevent false positives, a validated definition leveraging multiple sources of information that optimizes sensitivity as well as specificity is needed to fully understand the impact of COVID-19 infections and related outcomes, particularly among populations highly susceptible to infection such as people experiencing homelessness.

### Electronic supplementary material

Below is the link to the electronic supplementary material.

Supplementary Material 1

## Data Availability

Due to the vulnerability of the study population and the sensitive nature of the collected data, ethical approval for this study requires that study data remains on secure servers. As such, data presented in this study are not publicly available. However, queries about the datasets or programming supporting this study can be directed to the Corresponding Author.

## Abbreviations

PCR test / RT-qPCR: Polymerase chain reaction test
RAT: Rapid antigen test
CDC: US Centers for Disease Control and Prevention
Anti-N: Antibodies to nucleocapsid protein
Anti-R: Antibodies to receptor-binding domain protein
Anti-S: Antibodies to spike protein trimer
RR: Relative ratio
RBD: Receptor-binding domain protein
Spike: Spike protein trimer
