# Supplementary material for: Enhancing detection of SARS-CoV-2 re-infections using longitudinal sero-monitoring: demonstration of a methodology in a cohort of people experiencing homelessness in Toronto, Canada
Source: BMC Infect Dis. 2024 Feb 2;24:125. doi: 10.1186/s12879-024-09013-9 (PMC10835952; doi:10.1186/s12879-024-09013-9)
Supplement: Supplementary file 1 — Supplementary Material 1 [file 12879_2024_9013_MOESM1_ESM.docx]

**Supplementary Materials**

**Supplement 1 – Method for identifying potential SARS-CoV-2 re-infection events using recurring serology data**

The table below outlines the procedure used to identify potential re-infection events in our recurrent serologic assay data, which measures IgG antibodies to spike protein trimer (‘S’ or ‘spike’), spike protein receptor-binding domain protein (‘R’), and nucleocapsid protein (‘N’). The method depends on two primary, interrelated assumptions derived from current SARS-CoV-2 antibody kinetics literature:

1. *Nucleocapsid protein (N) antibody levels do not increase again following a peak level after incident infection*
   - SARS-CoV-2 antibody kinetics literature describes an initial N rise (which varies in duration and amplitude), then a decrease of levels (at varying rates, but generally slower after ~6mths).^1-3^ Other than considering the normal fluctuation for that antibody (captured by its coefficient of variation [COV]), higher increases/decreases during the same infection event are not expected.
2. *After N begins to decrease, new increases to seropositive N above the COV are sufficient evidence of possible re-infection.*
   - This assumption leverages assumption A (that N won’t rise again after its initial peak). Where a downward trend in N has been established, any later increase beyond the COV is ‘unexplained’ (in our data, this is at least 1.2942*the initial value^4^).
   - A recent paper assessing change in anti-N as a means of improving identification of SARS-CoV-2 infection^5^ found that an increase only slightly above the coefficient of variation was the optimal level ensuring comprehensive capture of infection without including false positives, supporting the assumption that increases above COV is not an unreasonable assumption.
   - Furthermore, we know response to N is highly variable, particularly in highly vaccinated environments.^6-7^

Finally, a few key definitions about our serology data to orient the reader:

- The vast majority of our serology samples (~92%) were plasma or had both plasma and dried blood spot. Where both sample types were provided plasma results were prioritized.
- The seropositivity threshold for N is 0.396 RR for plasma samples or 0.642 RR for dried blood spot (DBS) samples at the primary dilution (0.0625μL/well for plasma samples and 2.5μL/well for DBS);^4^
- The COV for N (for both Plasma and DBS) is 29.42%; thus, increases above N RR*1.2942 represent increase above the COV
- Values above 2.0 RR are considered outside of the linear range, at which point increases can no longer be assessed as being truly above or below the COV;
- Unless otherwise stated, all samples were tested at the primary dilution. Where the primary dilution produces values above the linear range, a secondary dilution (0.0039μL/well for Plasma and 0.625 μL/well for DBS) may be used to assess change in RR, if this different dilution produces at least one of two values within the linear range.
- Where intervals are missing the next available interval is considered instead, using the exact same logic.

| **Step 1: Combine Plasma and DBS samples into a single set of values per participant/interval**  The *Ku-gaa-gii pimitizi-win study* collected Plasma and/or DBS samples for serology, depending on what the participant could provide. Because the RR scales for each of these data types are different, individuals who provide different serology types at different intervals must be treated differently than individuals who always provide one type across intervals. Thus, the samples must be reconciled into a single value per participant and interval.  Step 1a. If DBS and Plasma values are both present for a particular participant and interval, keep Plasma over DBS (Plasma is more prevalent and generally considered the more accurate of the two data types^4^);  Step 1b. Flag intervals where serology type differs from the previous interval; these intervals will follow a modified step 2;  Step 1c. Flag where consecutive intervals both have values outside of the linear range (values above 2 RR^4^); increases outside the linear range are not meaningful and will not be considered as possible re-infections   1. In these flagged situations, the process outlined in step B may be run using a lower dilution (0.0039μL for Plasma and 0.625 for DBS), but only if this lower dilution provides at least one value within the linear range. |
| --- |
| **Step 2: Assess N values after incident infection is identified**  Step 2a. Where N begins as seronegative (< 0.396 [Plasma] or < 0.642 [DBS]), as may occur particularly at baseline if infection was reported early in the pandemic:   1. If data type is consistent in both intervals (ie. both Plasma or both DBS) and the latter N RR is seropositive, then flag interval as a **Potential Re-Infection** 2. If data type differs at both intervals (e.g. N1=Plasma and N2=DBS), and the latter N is seropositive *for that data type*, then flag interval as a **Potential Re-Infection**   Step 2b. Where N begins as seropositive (>=0.396 for Plasma or >=0.642 for DBS) and the data type is consistent in both intervals (ie. both Plasma or both DBS):   1. Compare initial N RR (‘N1’) and latter N RR (‘N2’):    1. If N2 is lower than N1, a downward trend is established to consider against the next N RR values (‘N3’ through ‘N5’);    2. If N2 is greater than N1*[1.2942], then no downward trend has been established, and we will compare against N3 (as this increase might be a continuation of the first infection event). 2. Compare later N RR value (‘N3’) against N2:    1. If a downward trend had been established and N3 is greater than N2*[1.2942] then flag interval as a **Potential Re-Infection**    2. If a downward trend was not established and N3 is (again) greater than N2, we will compare against N4 (though unlikely, it remains possible that N increases from a single infection event for more than 6 months)    3. If a downward trend was not established and N3 is lower than N2, a downward trend is established to consider against the next N RR values (‘N4’ and ‘N5’) 3. Compare N4 and N5 values the same way as above   See *Figure A* (below table, for flowchart) |
| **Step 3: Assess R/S values to assess probability of re-infection event**  For all intervals flagged as being **Potential Re-infection**, review R and Spike R levels:   1. Where participants had no evidence of vaccination within 6 months of the flagged interval, re-classify depending on the following:    1. If R or S are also elevated, classify as **Probable Re-infection**.    2. If neither R or S are elevated and are stable, classify as **Possible Re-infection**.    3. If neither R or S are elevated and are decreasing, classify as **Indeterminate Re-infection.** 2. Where participants had evidence of vaccination within 6 months of the flagged interval, re-classify depending on the following:    1. If R or S are also elevated or stable, classify as **Possible Re-infection**.    2. If neither R or S are elevated and are decreasing, classify as **Indeterminate Re-infection.** |


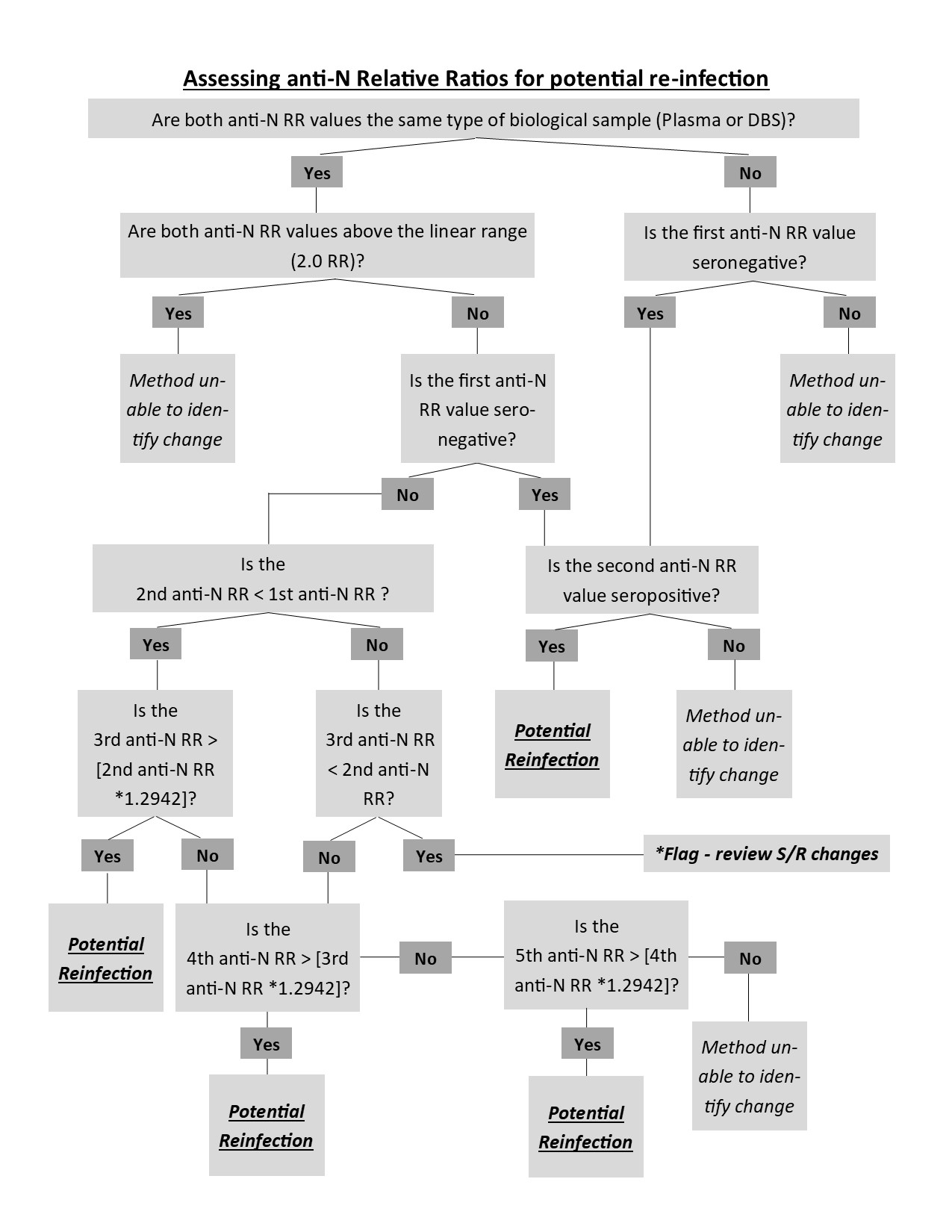


Figure S1 - Flowchart for step 2 of the serology method - assessing sequential anti-N relative ratio values

**Supplement 2 – Characteristics of participants at risk of re-infection during the observation period**

| **Participant demographics at baseline** | **Total (n=381)** |
| --- | --- |
| Age, mean (SD) | 46.65 (15.4) |
| Age category, N (%) |  |
| 16-29 years old | 48 (12.60%) |
| 30-49 years old | 169 (44.36%) |
| 50-69 years old | 133 (34.91%) |
| 70+ years old | 31 (8.14%) |
| Self-identified gender, N (%) |  |
| Male | 264 (69.29%) |
| Female | 112 (29.40%) |
| Non-binary/LGBTQ2S+ | 4 (1.05%) |
| Refused/Don’t know | 1 (0.26%) |
| Self-described race category, N (%) |  |
| White | 178 (46.72%) |
| Black | 102 (26.77%) |
| Other Racialized Groups or Multiracial | 90 (23.62%) |
| Refused/Don’t know | 11 (2.89%) |
| Citizenship status, N (%) |  |
| Citizen | 272 (71.39%) |
| Landed immigrant | 55 (14.44%) |
| Refugee claimant | 35 (9.19%) |
| Temporary/Other | 13 (3.41%) |
| Refused/Don’t know | 6 (1.57%) |
| Immigration history, N (%) |  |
| Immigrated to Canada > 10 years ago | 109 (28.61%) |
| Immigrated to Canada <= 10 years ago | 71 (18.64%) |
| Born in Canada | 201 (52.76%) |
| Top education completed, N (%) |  |
| Less than high school | 101 (26.51%) |
| High school diploma | 127 (33.33%) |
| Any post-secondary | 147 (38.58%) |
| Refused/Don’t know | 6 (1.57%) |
| Number of self-reported comorbidities^1^, N (%) |  |
| None | 204 (53.54%) |
| One | 100 (26.25%) |
| Two or more | 77 (20.21%) |
| Number of COVID-19 vaccine doses received by the baseline interview, N (%) |  |
| Zero | 83 (21.78%) |
| One | 70 (18.37%) |
| Two or more | 228 (59.89%) |

SD=Standard deviation

^1^ Comorbidities were diagnosed by a physician and self-reported by the participant, and include the following: hypertension; diabetes; asthma; chronic lung disease (including COPD, emphysema or chronic bronchitis); heart disease (including history of heart attack, heart failure, or coronary artery disease); history of stroke; chronic kidney disease; liver disease; chronic neurological disorder; cancer (active or historical); HIV/AIDS; and immune suppression (caused by conditions other than HIV/AIDS).

**References**

Movsisyan M., Chopikyan A., Kasparova I., *et al*. Kinetics of anti-nucleocapsid IgG response in COVID-19 immunocompetent convalescent patients. *Scientific Reports*. 2022; 12. Doi: 10.1038/s41598-022-16402-0

Loesche M., Karlson E., Talabi O., *et al*. Longitudinal SARS-CoV-2 Nucleocapsid Antibody Kinetics, Seroreversion, and Implications for Seroepidemiologic Studies. *Emerg Infect Dis*. 2022; 28(9): 1859-1862.

Van Eslande J., Oyaert M., Ailliet S., *et al*. Longitudinal follow-up of IgG anti-nucleocapsid antibodies in SARS-CoV-2 infected patients up to eight months after infection. *J of Clin Virology*. 2021; 136. Doi: 10.1016/j.jcv.2021.104765.

Colwill K., Galipeau Y., Stuible M., *et al.* A scalable serology solution for profiling humoral immune responses to SARS-CoV-2 infection and vaccination. *Clin Transl Immunology* **11**, e1380. Doi:10.1002/cti2.1380 (2022).

1. Bazin R., Rochette S., Perreault J., et al. Evaluation of anti-nucleocapsid level variation to assess SARS-CoV-2 seroprevalence in a vaccinated population. *Infectious Diseases*. 2023; 55 (6): 425-30.
2. Follmann D. JHE, Buhule O.D., Zhou H., Girard B., et al. Antinucleocapsid Antibodies After SARS-CoV-2 Infection in the Blinded Phase of the Randomized, Placebo-Controlled mRNA-1273 COVID-19 Vaccine Efficacy Clinical Trial. *Annals of Internal Medicine*. 2022;175:1258-65.
3. Whitaker B. GC, Otter A.D., Simmons R., et al. . Nucleocapsid antibody positivity as a marker of past SARS-CoV-2 infection in population serosurveillance studies: impact of variant, vaccination, and choice of assay cut-off. *medRxiv*. 2021.
